# Assessing related factors to fasting blood sugar and glycosylated hemoglobin in patients with type 2 diabetes simultaneously by a multivariate longitudinal marginal model

**DOI:** 10.1038/s41598-022-19241-1

**Published:** 2022-09-01

**Authors:** Samaneh Hosseinzadeh, Zahra Khatirnamani, Enayatollah Bakhshi, Alireza Heidari, Arash Naghipour

**Affiliations:** 1 Department of Biostatistics, University of Social Welfare and Rehabilitation Sciences, Tehran, Iran; 2 Health Management and Social Development Research Center, Golestan University of Medical Sciences, Gorgān, Iran

**Keywords:** Diseases, Risk factors, Mathematics and computing

## Abstract

The multivariate marginal model can be used to simultaneously examine the factors affecting both FBS and HbA1c using longitudinal data. The model fitted to multivariate longitudinal data should prevent redundant parameter estimation in order to have greater efficiency. In this study, a multivariate marginal model is used to simultaneously investigate the factors affecting both FBS and HbA1c with longitudinal data for patients with type 2 diabetes in Northern Iran. The present research is a retrospective cohort study. Overall, 500 medical records with complete information were reviewed. The multivariate marginal model is used to determine the factors associated with FBS and HbA1c using longitudinal data. Data have been analyzed in R-3.4.0 using ‘mmm2’ package. Given that the coefficients for the interactions of rtype with the intercept, time, family history of diabetes, history of hypertension, history of smoking, insulin therapy, systolic/diastolic blood pressure and duration of disease at first visit are significantly different from zero (P < 0.05), the effect of the independent variables on the two response variables is different and different coefficients should be used for each. Therefore, the interactions of these variables with rtype are kept in the final model. The coefficients for the interactions of rtype with sex, age at first visit, history of high cholesterol, and weight are not significantly different from zero (P > 0.05), indicating that their effect on the two response variables is similar and only one coefficient should be used for each. We examined the similarity of coefficients when fitting the longitudinal multivariate model for the relationship between FBS/HbA1c and sex, age, history of high blood cholesterol, and body weight. If an independent variable has similar effects on both responses, only one coefficient should be estimated, which will increase the efficiency of the model and the reliability of the results.

## Introduction

The increasing burden of non-communicable diseases (NCDs) is a major healthcare threat, especially in developing countries^1,2^. Type 2 diabetes is the most common form of diabetes among the different types of this disease (i.e., type 1 diabetes, gestational diabetes and diabetes mellitus) and accounts for about 90–95% of all diagnosed cases of diabetes^3^. According to the 2019 World Health Organization (WHO) report, about 422 million people are diagnosed with diabetes worldwide, the majority living in low- and middle-income countries^4^. Worldwide prevalence of diabetes is about 6.4%, and it is expected to reach 366 million by 2030^5^. In Iran, the prevalence of diabetes is about 10.3% in adults aged 20–74%, 9.6% in men, and 11.1% in women^6^. One study found that in 2018, endocrine, nutritional and metabolic diseases were the fifth leading cause of death in Golestan province^7^. Lost productivity, disability, and premature death from diabetes impose a substantial burden on society^8^.

Fasting blood sugar (FBS), glycolsylated hemoglobin (HbA1c) and Blood sugar 2-h postprandial (BS2hpp) are effective bi-omarkers in patients with diabetes. According to the National Diabetes Organization (NIDDK), glycosylated hemoglobin and fasting blood sugar indices are two available criteria for assessing and controlling a diabetic patient's condition, which helps physicians in prevent-ing its chronic complications^9^. The best way to control long-term diabetes is to measure gly-cosylated hemoglobin, which is a good tool for checking the patient's blood sugar and making treatment decisions, but it is not recommended for diagnosis^10^. Therefore, in this study, assessment and control of diabetic patients is based on FBS and HbA1c, which are measured and recorded in different time periods. Lower FBS and HbA1c levels are associated with reduced diabetes-related complications and morbidity, and testing of these factors during treatment is necessary.

Determining the factors associated with FBS and HbA1c is crucial to controlling and stabilizing them. Most studies have examined the factors affecting FBS and HbA1c levels using cross-sectional data and only consider one of these two variables^11,12^. However, FBS and HbA1c are linked and are affected by common factors^11^. In addition, the effect of these factors can vary over months and years. There have also been a number of longitudinal studies on only one of these two variables, with factors such as age, insulin therapy, and blood pressure being identified as factors influencing changes in FBS and HbA1c levels^13–16^.

While many questions can be answered by modeling different outcomes separately, some questions can only be answered in the combined analysis of all of them^17^. In the Raffa and Dubin study, which analyzed smoking cessation clinical trial data using a Bayesian method and the Markov Monte Carlo chain, the univariate response models, which were fitted separately, were compared with the two-variable response model. The efficiency of the two models was evaluated using confidence intervals, which were wider in the univariate model than in the multivariate model, that is, the model efficiency was higher in the multivariate model^18^.

However, the multivariate marginal model proposed by Asar and Ilk^19^ can be used to simultaneously examine the factors affecting both FBS and HbA1c using longitudinal data. This reduces the number of estimates, increases the degree of freedom for the error term, and enhances the efficiency of the model. Multivariate models are traditionally built on the assumption that relationships between the dependent and independent variables are separate, and estimate separate regression coefficients for each of these relationships. In many cases, however, all or some of these relationships are very similar. This leads to estimation of redundant regression coefficients and decreases the efficiency of parameter estimates. Therefore, the model fitted to multivariate longitudinal data should prevent redundant parameter estimation in order to have greater efficiency^19^.

In this study, a multivariate marginal model is used to simultaneously investigate the factors affecting both FBS and HbA1c with longitudinal data for patients with type 2 diabetes in Golestan Province, Northern Iran.

## Results

Of the 500 studied diabetic patients, 58.6% are female. The average age of the patients is 45.7 ± 8.9 years and the mean duration of the disease is 3.7 ± 2.7 years (0–15). 19.8% of the patients had a history of smoking, 64.5% had a family history of diabetes, 61.7% had a history of high cholesterol, and 70.4% had a history of high blood pressure. 14.6% of patients used insulin during period under study (Table 1).

**Table 1:** Destribution of the patients’ characteristics.

Qualitative Variables	Levels	N (%)
Gender	Male	207 (41.4)
	Female	293 (58.6)
History of smoking	Yes	99 (19.8)
	No	401 (80.2)
Family history of diabetes	Yes	322 (64.5)
	No	178 (35.5)
History of high blood cholestrol	Yes	308 (61.7)
	No	192 (38.3)
History of hypertension	Yes	352 (70.4)
	No	148 (29.6)
Insulin therapy	Yes	95 (14.6)
	No	405 (85.4)

Quantitative Variables	Mean (SD)	
Measurement time (months)	16.2 (11.3)	
Age at first visit (years)	45.7 (8.9)	
Duration of disease at first visit (years)	3.7 (2.7)	
Weight at first visit (kg)	78.0 (12.6)	
Systolic blood pressure at first visit (mg/dl)	138.8 (17.6)	
Diastolic blood pressure at first visit (mg/dl)	80.3 (7.7)	

Changes in FBS and HbA1c show a downward trend. The mean FBS level decreased from about 308 to 163 mg/dl at baseline, and the mean HbA1c level decreased from about 9.6% to 7% (Table 2). The correlation between FBS and HbA1c is significant and positive in all measurement times (P-value < 0.001). Pearson correlation coefficient ranges from 0.61 to 0.76 (Table 2).

**Table 2:** Correlation, mean, and standard deviation of FBS and HbA1c in patients by measurement time.

Measurement time	FBS		HbA1c		Correlation coefficient (FBS and HbA1c)
	Mean (SD)	> 126 mg/dl (%)	Mean (SD)	> 6.5% (%)	
Baseline	307.9 (58.7)	99.6	9.6 (1.0)	99.8	0.61
3	278.1 (52.8)	99.8	9.1 (1.1)	100.0	0.61
6	257.5 (50.9)	99.2	8.6 (1.0)	99.4	0.64
9	242.8 (47.7)	98.8	8.4 (0.9)	99.8	0.61
12	239.0 (49.8)	99.2	8.3 (0.9)	99.4	0.61
15	230.3 (50.7)	98.3	8.1 (0.9)	97.7	0.69
18	230.2 (52.2)	97.3	8.1 (0.8)	97.8	0.69
21	220.4 (51.8)	96.4	7.9 (0.8)	96.6	0.68
24	215.1 (60.6)	92.4	7.8 (0.9)	92.1	0.74
27	206.5 (59.4)	93.1	7.7 (0.9)	78.1	0.72
30	205.9 (63.7)	89.5	7.7 (0.9)	89.1	0.72
33	19.4 (61.5)	80.3	7.5 (0.9)	84.6	0.72
36	177.8 (58.6)	77.7	7.3 (0.9)	79.5	0.72
39	163.1 (51.2)	69.2	7.0 (0.8)	68.7	0.73
Total	235.4 (65.0)	94.7	8.2 (1.1)	94.6	0.76

A univariate model is used to determine the correlation structure of dependent variables over time, and several correlation structures are examined and compared to the Quasi-likelihood under Independence Model Criterion (QIC). Among different correlation structures, ‘Exchangeable’ has the lowest QIC. Therefore, this correlation structure is used in the multivariate model.

First, all the independent variables as well as their interactions with the indicator variable $rtype$ are entered into the model. Given that the coefficients for the interactions of $rtype$ with the intercept, time, family history of diabetes, history of hypertension, history of smoking, insulin therapy, systolic/diastolic blood pressure and duration of disease at first visit are significantly different from zero (P < 0.05), the effect of the independent variables on the two response variables is different and different coefficients should be used for each. Therefore, the interactions of these variables with $rtype$ are kept in the final model. The coefficients for the interactions of $rtype$ with sex, age at first visit, history of high cholesterol, and weight are not significantly different from zero (P > 0.05), indicating that their effect on the two response variables is similar and only one coefficient should be used for each. These interactions are removed from the final model.

Table 3 reports the results of fitting the model to the Exchangeable correlation structure. In this table, the intercepts for both FBS (P < 0.001) and HbA1c (P = 0.004) responses are significant. The effect of time on changes in FBS and HbA1c levels is negative (P < 0.001), meaning that FBS levels have decreased over time, with an average 3.55 mg/dl decrease in FBS per month. In addition, HbA1c levels have decreased by an average of 0.15% per month. The effect of family history on FBS is significant and negative (P = 0.003); that is, average FBS level during follow-up is 8.97 mg/dl lower in people with a family history of diabetes than others. The effect of family history of diabetes on HbA1c is not significant (P = 0.108). The effect of having a history of hypertension on FBS is significant and negative (P = 0.038) as average FBS level during follow-up is 8.67 mg/dl lower in patients with a history of hypertension than others. The effect of family history of hypertension on HbA1c is not significant (P = 0.340). The effect of smoking on FBS is significant and positive (P = 0.006) as average FBS level during follow-up is 11/20 mg/dl higher in smokers than in non-smokers. The effect of smoking on HbA1c is not significant (P = 0.369). The effect of insulin therapy on FBS is significant and positive (P < 0.001) as the average FBS level during follow-up is 25.98 mg/dl in people who received insulin therapy than those who did not. The effect of insulin therapy on HbA1c is also significant and positive (P = 0.021) as average HbA1c level during follow-up is 37.5% higher in people who received insulin therapy than in those who did not. The effect of systolic blood pressure on FBS (P < 0.001) and HbA1c (P = 0.006) is significant during follow-up. Average FBS level is 0.54 mg/dl higher in people with higher systolic blood pressure than those with lower systolic blood pressure. Also, average HbA1c level is 12% lower in people with higher systolic blood pressure than those with lower systolic blood pressure. The effect of duration of infection on arrival at FBS is significant (P < 0.001), indicating that, with all conditions remaining constant, average FBS level has decreased by 3.09 mg/dl per year. Diastolic blood pressure, sex, age at first visit, weight, and history of high cholesterol have no significant effect on FBS and HbA1c during follow-up (P > 0.05).

**Table 3:** Results of fitting Model III (final model) for estimating regression coefficients.

Variables	FBS				HbA1C			
	coefficient	SE	z-value	P-value	Coefficient	SE	z-value	P-value
Intercept	198.02	15.16	13.07	< 0.001	26.73	9.97	2.68	0.004
Time	− 3.05	0.09	− 32.25	< 0.001	− 0.15	0.03	− 5.45	< 0.001
Family history of diabetes	− 8.97	3.31	− 2.71	0.003	0.58	0.47	1.24	0.108
History of hypertention	− 8.67	4.87	− 1.78	0.038	0.64	1.54	0.41	0.340
History of smoking	11.20	4.42	2.54	0.006	0.46	1.36	0.34	0.369
Insulin therapy	25.98	5.60	4.64	< 0.001	5.37	2.64	2.03	0.021
Systolic blood pressure	0.54	0.11	5.08	< 0.001	− 0.12	0.05	− 2.50	0.006
Diastolic blood pressure	0.27	0.17	1.55	0.061	− 0.10	0.08	− 1.35	0.089
Duration of disease	− 3.09	0.94	− 3.27	< 0.001	− 0.15	0.28	− 0.52	0.300
Sex (male)	1.76	1.88	0.94	0.174	1.76	1.87	0.94	0.174
Age at first visit	− 0.11	0.11	− 0.99	0.161	− 0.11	0.11	− 0.99	0.161
History of high blood cholestrol	2.18	1.92	1.13	0.129	2.18	1.92	1.13	0.129
Weight at first visit	0.13	0.08	1.58	0.057	0.13	0.08	1.58	0.057

## Discussion

This study investigated the factors associated with changes in FBS and HbA1c levels over time using a multivariate margin model with longitudinal data from 500 patients with type 2 diabetes. First, the model was fitted to the data with different correlation structures in univariate analysis. The ‘Exchangeable’ structure in the model with the lowest QIC was used in multivariate analysis. In multivariate analysis, first all the independent variables and their interactions with the indicator variable $rtype$ were entered into the model, and the significance of the interaction coefficients was tested. Then, the final model was formulated with the most efficient combination of coefficients.

The results showed that there was a significant positive relationship between FBS and HbA1c during follow-up. This correlation was studied using multivariate models which allow for examining the factors associated with changes in FBS and HbA1c over time. Similarly, Simon et al. showed that for one percent increase in HbA1c, FBS increases by 30 mg/dl^11^. This correlation highlights the diagnostic value of FBS in disease control and follow-up for patients for whom measuring HbA1c is not possible.

In the multivariate model, measurement time, family history of diabetes, history of hypertension, history of smoking, insulin therapy, systolic blood pressure, and duration of disease at first visit were significantly associated with changes in FBS levels, while measurement time, insulin therapy, and systolic blood pressure were significantly associated with changes in HbA1 levels.

The results also showed that FBS and HbA1c levels had a downward trend over three years (based on the effect of time on the model). This suggests that the condition of patients with type 2 diabetes has been under control and is improving. Other cross-sectional^11^ and longitudinal^2,13^ studies have shown that HbA1c is related to how individuals visit health centers. In one study, Van Casteren et al.^20^ showed that the quality of care for patients covered by the diabetes care scheme in Belgium was higher and positive trends were observed in disease outcomes. Chang et al.’s^21^ longitudinal study of diabetes management program in Taiwan showed that strategies beyond the existing education and pharmacotherapeutic schemes are needed to lower HbA1c. In the longitudinal study of Tan et al.^13^ that covered more than three years, a slight decrease in HbA1c was observed in the initial 3 years, but it increased in the following 2 years. According to the American Diabetes Association, the goal of nutrition therapy and exercise is to improve overall health, achieve proper weight, attain glycemic, blood pressure and lipid goals, and delay the complications of diabetes such as cardiovascular disease^22^. Although the present research does not provide details of the diet and exercise regimen for each person, it is clear that if a minimum of these services are provided and the patient follows them, they are still effective and help reduce patients’ blood sugar levels.

Family history of diabetes was identified as a factor associated with changes in FBS. People with a family history of diabetes had lower FBS levels than others during follow-up. This may be due their familiarity with methods of disease prevention and control as well as its complications, which enabled them to achieve better health outcomes. Most studies investigate the effect of family history of diabetes on the incidence of this disease^23,24^. Moreover, in a 2014 longitudinal study by Wen et al.^14^ which examined the trend of HbA1c changes, family history was not found to be significantly related.

In the present research, smoking was identified as one of the factors associated with FBS changes, with non-smokers having better blood sugar control during follow-up. In a 2016 cross-sectional study by Junia et al.^12^, smoking was identified as a factor in insulin resistance and type 2 diabetes, and they found that hypoglycemia was more severe in smokers than non-smokers. Other longitudinal studies have confirmed the relationship between smoking and poor blood sugar control in diabetics^25–27^.

Our results showed that the relationship between insulin therapy and changes in FBS and HbA1c was significant. That is, mean changes in FBS and HbA1c were higher in people who used insulin than those who did not use insulin. Chen et al., conducted a longitudinal observational study in Taiwan in which all the studied patients used insulin. The results indicated significant reduction in FBS and HbA1c in patients with type 2 diabetes who used insulin^15^. There are no studies that compare changes in FBS and HbA1c in insulin and non-insulin groups. It is recommended that patients who have been prescribed insulin therapy be more closely monitored and that doses be adjusted to each patient’s condition during treatment.

The history of hypertension was another factor associated with changes in FBS, with the extent and speed of decrease in FBS being significantly higher in people without a history of hypertension. This suggests that the disease is more difficult to control in people with high blood pressure. In addition, there was a significant positive relationship between systolic blood pressure and changes in FBS and HbA1C. Similarly in the longitudinal study of Kazemi et al.^16^, a significant relationship was observed between blood pressure and increase in HbA1c. Lowering blood pressure can help control diabetes and prevent its complications, but it should be done carefully to achieve the desired results.

In this study, no significant relationship was observed between body weight (body mass index) and changes in FBS and HbA1C over time. On the other hand, the duration of the disease was identified as a factor associated with changes in FBS. It is universally accepted that weight loss is very important for diabetic patients, and patients often lose weight during diabetes control. The lack of a significant relationship between body weight and FBS/HbA1C in the present research may be due to the duration of the disease. More specifically, the average duration of the disease at the time of admission was about 2.5 years, while weight loss may have mostly occurred at the onset of the disease. There was no significant relationship between the patient’s age at first visit and changes in FBS/HbA1c. In a cross-sectional study, Danai et al.^28^ found no relationship between age and changes in HbA1c. However, the longitudinal study of Chang et al.^14^ confirmed the relationship between age and changes in HbA1c. Using a logistic regression model, the cross-sectional study by Quah et al.^29^ showed that HbA1c is more likely to be higher in younger adults. Another study found that older people were more likely to adhere to their doctor’s prescriptions, resulting in a further reduction in complications^30^. In the present research, no significant relationship was observed between sex and changes in FBS/HbA1c. Similarly, the cross-sectional studies of Ismaili Nasab et al.^31^ and Quah et al.^29^ and the longitudinal study of Wen et al.^14^ showed that the two are not significantly related. History of high cholesterol was not significantly associated with changes in FBS and HbA1c. A number of other studies have also found no significant relationship between blood lipids and FBS/HbA1c control^2,27^.

Managing blood sugar is very important for preventing or delaying acute and long-term complications of diabetes. A clinical trial by Pourverdi et al.^32^ found that individual management of diabetes lowers blood sugar level, reduces HbA1C, and improves the overall health of patients with type 2 diabetes.

### Limitations

Diabetes is a chronic condition in which adaptation, modulation of biomarkers, and control and management of diet and medication play a high principal role in the survival of the individual and increase the quality of life of the patient^33^. Studies have shown that exercise significantly reduces the concentration of C-reactive protein, triglycerides, systolic and diastolic blood pressure, HbA1c, and insulin resistance^34,35^. In other studies, a significant direct relationship was observed between the prevalence of sleep disorders in people with diabetes and the increase in HbA1c. Sleep disorders may be a risk factor for increased HbA1c levels^36,37^. Social capital also plays a principal role in the prevention, treatment, and care of diabetes. Numerous other studies show that social capital has a protective effect in reducing stressful situations, reducing high-risk behaviors, reducing depression and psychological disorders, reducing mortality, and improving overall health^38,39^. Therefore, the most principle limitation of the present study was its retrospective; Hence, the drawbacks of retrospective studies also apply to this study. Interventions such as diet, exercise, sleep, family structure, and social life can be effective factors affecting FBS and HbA1c that were not fully recorded in their medical files. Also, the BS2hpp, despite its great importance in estimating HbA1c due to lack of consistency in patients' records, was not included as a response variable in this study.

It is recommended to use other statistical methods such as trajectory framework to study the changes in FBS and HbA1C in patients with type 2 diabetes with a follow period of more than three years, since the course of the disease may change completely after three years.

## Conclusion

We examined the similarity of coefficients when fitting the longitudinal multivariate model for the relationship between FBS/HbA1c and sex, age, history of high blood cholesterol, and body weight. If an independent variable has similar effects on both responses, only one coefficient should be estimated, which will increase the efficiency of the model and the reliability of the results.

## Methods

The present research is a retrospective cohort study. The statistical population consisted of patients with type 2 diabetes covered by the “National Program for Prevention and Control of Diabetes (NPPCD)” who had referred to health centers across Kordkuy city in Golestan Province between 2013 and 2016. Inclusion criteria were: (1) diagnosis with type 2 diabetes; (2) at least two visits to the health center; and (3) availability of complete medical records. Health centers were selected using cluster random sampling. That is, the health centers were divided into several clusters, but due to the similarity of these centers and of the culture in different areas of the city, five health centers were randomly selected and 100 cases were systematically sampled from each center. Overall, 500 medical records with complete information were reviewed. The information recorded on Diabetes Patient Care Flow Sheets included demographic and contextual factors such as sex, age at first visit, family history of diabetes, history of high blood cholesterol, history of smoking, history of hypertension, and duration of the disease. Clinical examination results included systolic blood pressure, diastolic blood pressure, and patient weight along with the results of blood tests (e.g., FBS and HbA1c) performed every three months. Therefore, FBS and HbA1c are considered as the dependent variables and the other factors as the independent variables. Related information was extracted from the patients’ medical records in a checklist that had been prepared for this purpose.

This research has been approved by the National Committee for Ethics in Biomedical Research. The informed consent was waivered by the National Committee for Ethics in Biomedical Research (Ethics code: IR.USWR.REC.1395.239) because patient information was anonymized. The authors confirm that all methods were carried out in accordance with relevant guidelines and regulations.

### National Program for Prevention and Control of Diabetes (NPPCD)

NPPCD was a clinical trial initiated in February 1998 in 17 Iranian medical universities and continued until 2002 for people over 30 years old and pregnant women. It was revised in 2002 and has been actively implemented throughout the country since 2004. The general goals of NPPCD are early diagnosis, appropriate care, and treatment of diabetes complications^40,41^. In this program, patients with type 2 diabetes are monitored by a resident physician in health centers after being diagnosed and consenting to treatment, and receive the necessary recommendations regarding diet and exercise. In addition, the necessary tests are performed once every three months and the results of these and other clinical examinations are recorded in the patient’s file (Diabetes Patient Care Flow Sheets).

### Data analysis

The multivariate marginal model proposed by Asar and Ilk^19^ is used to determine the factors associated with FBS and HbA1c using longitudinal data. In this model, FBS and HbA1c are defined as quantitative dependent variables, and each independent variable can have separate effects on the two response variables. According to this approach, some factors have similar effects on both dependent variables, and thus the indicator variable ‘response type’ ($rtype$) is included in the model to reduce redundant coefficients and increase the efficiency of parameter estimation. This variable takes the value of 0 and 1 (in this study, the dependent variable FBS is assumed to be 0 and the dependent variable HbA1c is assumed to be 1), and it is interacted with each of the independent variables in the model. With these assumptions and to fit the data related to patients with type 2 diabetes, model (1) is formulated as follows:

1 $$\begin{aligned} g\left( {Y_{{it_{j} }} {|}X_{{it_{j} }} } \right) & = \beta_{0} + \left( {\beta_{1} Time_{it} } \right) + \left( {\beta_{2} X_{1} } \right) + \left( { \beta_{3} X_{2} } \right) + \left( {\beta_{4} rtype_{j} } \right) + \left( {\beta_{5} \left( {time_{it} \times rtype_{j} } \right)} \right) \\ & \;\; + \beta_{6} \left. {\left( {X_{1} \times rtype_{j} } \right)} \right) + \left( {\beta_{7} \left( {X_{2} \times rtype_{j} } \right)} \right) \\ \end{aligned}$$

where $X_{1}$ and $X_{2}$ are the independent variables and $Time_{it}$ denotes the time of measurement of the dependent variables.

Assuming that the $rtype$ for the response variable FBS is zero, the model can be expressed as follows:

2 $$g \left( {Y_{{it_{1} }} {|}X_{{it_{1} }} } \right) = \beta_{0} + \left( {\beta_{1} Time_{it} } \right) + \left( {\beta_{2} X_{1} } \right) + \left( {\beta_{3} X_{2} } \right)$$

Assuming that $rtype$ for the response variable HbA1c is one, the model can be expressed as follows:

3 $$g \left( {Y_{{it_{2} }} {|}X_{{it_{2} }} } \right) = \left( {\beta_{0} + \beta_{4} } \right) + \left( {\left( {\beta_{1} + \beta_{5} } \right) \times time_{it} } \right) + \left( {\beta_{2} + \beta_{6} } \right)X_{1} + \left( {\beta_{3} + \beta_{7} } \right)X_{2}$$

In the two models above, the intercepts and the coefficients for the effects of the independent variables on the dependent variables FBS and HbA1C are different. The intercept for the FBS response is $\beta_{0}$ and the intercept for the HbA1C response is $\beta_{0} + \beta_{4}$. Also, coefficient for the effect of time on FBS is $\beta_{1}$ and on HbA1C is $\beta_{1} + \beta_{5}$.

After estimating the coefficients, we check for the similarity of the effect of each independent variable on the two dependent variables (i.e., redundant estimation) by examining whether the coefficients for the interaction of the independent variable and the indicator variable are zero (e.g., $\beta_{6}$ for the independent variable $X_{1}$). If the interaction coefficients are not significant (i.e., equal to 0), the relationship between the independent variable and the two dependent variables is similar (equal to $\beta_{2}$ in this example); therefore, it is not necessary to calculate two separate estimates for each dependent variable and one coefficient is sufficient. This reduces the number of coefficients (parameters) and increases the efficiency of the model. In other words, a more efficient model (i.e., a model with fewer parameters) is estimated. A generalized estimating equation (GEE) is used to estimate the parameters in this model^19^. Three steps were done to fitted final model.

- Step I: Fitting the initial with all the independent variables.
- Step II: Fitting a more efficient model by removing independent variables interaction with $rtype$ is not significantly different from 0. If the null hypothesis is accepted for the coefficients of terms multiplied by $rtype$ ($\beta_{s} \times X_{i} \times rtype_{j} , s = 5, \ldots ,7$), these terms are removed from the model.
  4 $$\begin{aligned} Yit & = \beta_{0} + \left( {\beta_{1} \times time_{it} } \right) + \left( {\beta_{2} \times X_{i1} } \right) + \beta_{3} \times X_{2} + \left( {\beta_{4} \times rtype_{j} } \right) + \left( {\beta_{5} \times timeit \times rtype_{j} } \right) + \left( {\beta_{6} \times X_{i1} \times rtype_{j} } \right) \\ & \;\; + \left( {\beta 7 \times X_{i12} \times rtype_{j} } \right) \left( {t = 1, \ldots ,ni} \right)\left( {j = 1, \ldots ,k} \right) \left( {i = 1, \ldots ,N} \right) { } \\ \end{aligned}$$
- Step III: The final multivariate model where the effect of independent variables on each of the dependent variables is estimated.

Data have been analyzed in R-3.4.0 using ‘mmm2’ package. A significance level of 0.05 is considered in all the analyses.

## Data Availability

The datasets generated and/or analyzed during the current study are not publicly available but are available via communication with the senior author (ZK) on reasonable request.
